# Advanced Basal Cell Carcinoma in a Geriatric Patient: A Case Report and Ethical Considerations

**DOI:** 10.7759/cureus.108326

**Published:** 2026-05-05

**Authors:** Luis Velázquez Arenas, Sarahi Garay Enriquez, Daniela Gómez Guerra

**Affiliations:** 1 Dermatology, School of Medicine and Health Sciences at Tecnológico de Monterrey, Monterrey, MEX; 2 Medicine, School of Medicine and Health Sciences at Tecnológico de Monterrey, Monterrey, MEX

**Keywords:** adverse effects, basal cell carcinoma, locally advanced basal cell carcinoma, skin cancer, vismodegib

## Abstract

Basal cell carcinoma (BCC) is the most common malignancy worldwide and is typically associated with favorable outcomes. However, a small subset progresses to locally advanced disease with limited therapeutic options. Here, we report a case of an 83-year-old man who presented with an unresectable scalp lesion treated with vismodegib, who developed liver dysfunction followed by rapid progression to multiple organ failure and death. This case highlights the challenges of managing advanced BCC in elderly patients and the need for careful risk-benefit assessment when considering systemic therapy.

## Introduction

Basal cell carcinoma (BCC) represents the most common cancer worldwide, with approximately 3.6 million new cases diagnosed annually in the United States. Its development is strongly associated with fair skin phototypes, age over 50 years, and cumulative ultraviolet radiation exposure. Additionally, molecular alterations, particularly dysregulation of the sonic hedgehog signaling pathway, which plays a critical role in cell growth and differentiation, contribute to its pathogenesis [1]. 

BCC generally carries an excellent prognosis, as most tumors are successfully treated with local excision or other standard therapies and are associated with low recurrence rates [1]. Nevertheless, a small proportion of cases progress to advanced disease, which includes either locally advanced (laBCC) or metastatic BCC [2]. LaBCC accounts for 0.8% of all BCC cases and refers to primary or recurrent tumors that are typically large and aggressive, and cannot be effectively managed with curative surgery or radiotherapy, often requiring a multidisciplinary approach [2,3]. 

Here, we present a case of an 83-year-old man with a 30-year history of an untreated, laBCC. Following multidisciplinary assessment, surgical resection and radiotherapy were deemed unsuitable, and systemic vismodegib was initiated; treatment-related complications ultimately resulted in the patient’s death. Available therapeutic options and relevant ethical considerations are discussed.

## Case presentation

**Figure 1:**
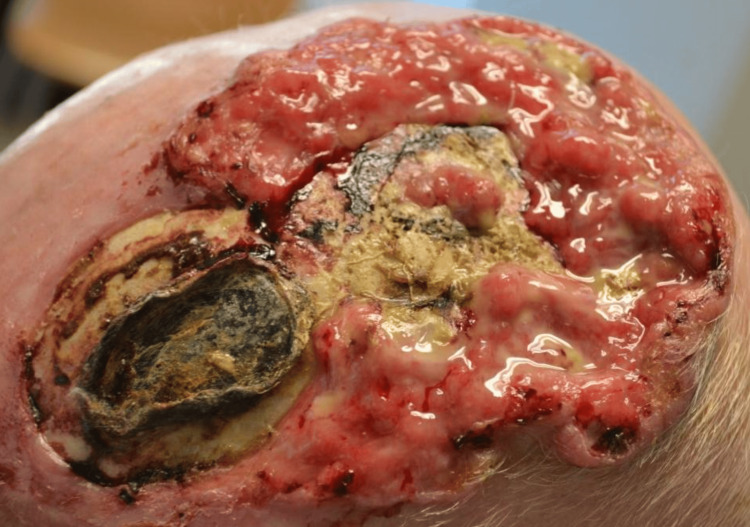
Locally advanced basal cell carcinoma of the scalp.

An 83-year-old man with a history of chronic occupational sun exposure and no relevant past medical history presented for evaluation of an ulcerated neoplasm in the parietal region of the scalp. The lesion had a 30-year evolution and measured approximately 10 x 15 cm; the patient had not received any previous medical treatment (Figure 1). On physical examination, an ulcerated nodular tumor was observed, with pearly, raised borders and telangiectasias on the peripheral surface. There was deep involvement with exposure and invasion of the parietal bone. The lesion was painless, with a fetid odor and purulent discharge. Histopathological examination confirmed the diagnosis of infiltrative BCC with bone involvement. Initial laboratory studies were within normal limits. Magnetic resonance imaging showed no evidence of intracranial extension. Chest radiography revealed no findings suggestive of pulmonary metastasis, and abdominal computed tomography did not identify masses or signs of distant dissemination. The case was evaluated in a multidisciplinary manner by the plastic surgery, neurosurgery, and radiology services, all of which declined both surgical intervention and radiotherapy, opting instead for conservative management. In this context, treatment was managed exclusively by the dermatology service, and systemic therapy with vismodegib was initiated. Treatment had been recently started, with less than one month of exposure, when the patient developed abnormalities in liver function tests, followed by rapid progression to multiple organ failure. The subsequent evolution was unfavorable, ultimately resulting in the patient’s death.

## Discussion

Mohs micrographic surgery remains the treatment of choice for high-risk, recurrent, or large BCC, particularly in anatomically challenging locations [4]. However, in advanced stages, surgical management may be limited by the inability to achieve clear margins and the potential for substantial morbidity. In contrast, radiotherapy may represent an alternative for patients who are not surgical candidates; however, its utility may be limited in specific scenarios, particularly in the presence of bone or cartilage invasion, as observed in the present case. In these settings, Hedgehog pathway inhibitors have emerged as an important systemic therapeutic option.

Currently, vismodegib and sonidegib are approved for clinical use, with vismodegib being the most widely utilized agent worldwide [5]. Nevertheless, their use is frequently associated with the presentation of adverse events. In the STEVIE trial, 98% of patients receiving vismodegib experienced at least one adverse event, and among them, 31% discontinued treatment. The most commonly reported effects included muscle spasms, alopecia, dysgeusia, weight loss, and fatigue [6]. Although most adverse events associated with vismodegib are non-life-threatening, hepatotoxicity has also been described as an infrequent yet potentially severe complication. Postmarketing surveillance data reported by Edwards et al. identified 94 cases with at least one manifestation of hepatic dysfunction in the US Food and Drug Administration Adverse Event Reporting System between 2009 and 2015. Of these, 34 were categorized as severe hepatotoxicity, with 20 cases resulting in hospitalization or death [7,8]. Notably, progression to multiple organ failure during vismodegib therapy appears to be an uncommon and severe occurrence, underscoring the unusual clinical course observed in the present case; nevertheless, given the single-case design, a direct causal association cannot be determined.

Beyond its clinical implications, this case also brings into focus the ethical complexity inherent to the management of laBCC in geriatric patients. Therapeutic decisions should integrate key geriatric principles, including life expectancy, functional status, comorbidity burden, and the anticipated lag time to benefit, to determine whether a given intervention is likely to confer meaningful clinical advantage within the patient’s remaining lifespan. In this context, shared decision-making becomes essential, incorporating not only the physician’s clinical judgment but also the patient’s values, preferences, and psychosocial context, with the active participation of family members and caregivers when appropriate [9].

## Conclusions

This case highlights the significant therapeutic and ethical challenges involved in the management of unresectable locally advanced BCC in elderly patients. Although Hedgehog pathway inhibitors, such as vismodegib, provide an important systemic alternative when surgery and radiotherapy are not feasible, clinicians must remain aware of the potential for severe and even fatal adverse events. Careful multidisciplinary evaluation, close monitoring during treatment, and individualized shared decision-making are essential for optimal management of laBCC. It is important to acknowledge the inherent limitations of the present case and that additional reports are required to better characterize any potential causal relationship between vismodegib therapy and multiple organ failure.
